# 

**Published:** 1839-04

**Authors:** 


### CONTENTS OF No. XIV.

OF THE

### British and Foreign Medical Review.

APRIL, 1839.

---

### Part First.—Analytical and Critical Reviews.

PAGE

Art. I.—1. Mémoire sur la Découverte du Magnétisme Animal. Par A. Mesmer 301
Memoir on the Discovery of Animal Magnetism. By A. Mesmer
2. Ueber den thierischen Magnetismus in einem Briefe an Hoffmann, (mit Zusätzen.) Von E. Gmelin . . . . . ib.
On Animal Magnetism, in a Letter to Hoffmann, (with Appendices.) By E. Gmelin.
3. A Practical Display of the Philosophical System called Animal Magnetism . ib.
4. ΑΣΚΛΗΠΙΕΙΟΝ. Allgemeines medicinisch-chirurgisches Wochenblatt für alle Theile der Heilkunde und ihrer Hülfswissenschaften . . ib.
ΑΣΚΛΗΠΙΕΙΟΝ. General Medico-chirurgical Weekly Journal for all Branches of Medicine, and its collateral Sciences.
5. Versuch einer Darstellung des animalischen Magnetismus als Heilmittel. Von C. F. A. Kluge . . . . . . ib.
Essaytowards a History of Animal Magnetism as a Remedy. By C. F. A. Kluge.
6. Jahrbücher für den Lebensmagnetismus, oder Neues Askläpieion. Allgemeines Zeitblatt für die gesammte Heilkunde nach den Grundsätzen des Mesmerismus; herausgegeben von Dr. K. C. Wolfart . . . ib.
Annals of Vital Magnetism, or New Ασκληπιειον. General Journal of Medical Science according to the Principles of Mesmerism. By Dr. K. C. Wolfart.
7. System des Tellurismus, oder thierischen Magnetismus. Ein Handbuch für Naturforscher und Aertze. Von Dr. D. G. Kieser. . . 302
System of Tellurism, or Animal Magnetism: a Manual for Philosophers and Physicians. By Dr. D. G. Kieser.
8. Du Magnétisme Animal en France, et des Jugements qu'en ont portés les Sociétés savantes, avec le Texte des divers Rapports faites en 1784, par les Commissaires de l'Académie des Sciences de la Faculté, et de la Société Royale de Médecine, et une Analyse des dernières Séances de l'Académie Royale de Médecine, et du Rapport de M. Husson; suivi de Considérations sur l'Apparition de l'Extase, dans les Traitemens Magnétiques. Par A. Bertrand. ib.
On Animal Magnetism in France, and on the Judgments pronounced on it by the learned Societies, with the Text of the different Reports made in 1784, by the Commissioners of the Academy of Sciences, and of the Faculty, and of the Royal Society of Medicine, and an Analysis of the Report of M. Husson; followed by Considerations on the Delirium observed in Treatment by Magnetism. By Alexander Bertrand.
9. Die Seherin von Prevorst. Eröffnungen über das innere Leben des Menschen und über das Hereinragen einer Geisterwelt in die unsere. Mitgetheilt von Justinus Kerner . . . . . . ib.
The Prophetess of Prevorst. Revelations on the inner Life of Man, and on a World of Spirits extending into our Sphere. By Justinus Kerner.
10. Heilungen durch Animalischen Magnetismus bewirkt. Herausgegeben von Dr. J. Bork . . . . . . . ib.
Cures performed by Animal Magnetism. Edited by Dr. J. Bork.
11. An Introduction to the Study of Animal Magnetism. By Dupotet de Sennevoy. ib.
12. Fingerzeige Gottes, in göttlichen Offenbarungen für einer Somnambulen himmlisches und irdisches Heil. (Der Ertrag ist für einen wohlthätigen Zweck bestimmt.) . . . . . . ib.
Hints from God, in Revelations for the Celestial and Terrestrial Salvation of a Somnambulist. (The profits to be devoted to charity.)
13. The Lancet and Medical Gazette. Volumes for 1837 and 1838. . . ib.

PAGE

ART. II.—Recherches pratiques sur l'Inspection et la Mensuration de la Poitrine, considérées comme Moyens diagnostiques complémentaires de la Percussion et de l'Auscultation. Par EUG. J. WOILLEZ, M.D. . . . 353
Practical Researches on the Inspection and Mensuration of the Chest, considered as complementary of Percussion and Auscultation. By E. J. WOILLEZ, M.D.

ART. III.—Intermarriage: or, the Mode in which, and the Causes why, Beauty, Health, and Intellect result from certain Unions; and Deformity, Disease, and Insanity from others, &c. By ALEXANDER WALKER . . 370

ART. IV.—1. Autoplastie, ou Restauration des parties du corps qui ont été détruites, à la faveur d'un emprunt fait à d'autres parties plus ou moins éloignées. Par PH. F. BLANDIN . . . . . . . 386
Autoplastic Surgery, or the Restoration of parts of the Body which have been destroyed, by borrowing from other parts more or less distant. By P. F. BLANDIN.
2. Handbuch der plastischen Chirurgie. Von Dr. EDWARD ZEIS . . ib.
Manual of Plastic Surgery. By Dr. EDWARD ZEIS.
3. Chirurgische Erfahrungen, besonders über die Wiederherstellung zerstörter Theile des menschlichen Körpers nach neuen Methoden. Von J. F. DIEFFENBACH . . . . . . . ib.
Chirurgical Essays, more particularly on the Restoration of lost Parts by new methods. By J. F. DIEFFENBACH.
4. Practical Surgery. By ROBERT LISTON. . . . . . ib.

ART. V.—1. The Works of JOHN HUNTER, F.R.S.; with Notes by J. F. PALMER 417
2. A Treatise on Inflammation. By JAMES MACARTNEY, M.D. F.R.S. F.L.S. &c. . ib.
3. Illustrations of the Elementary Forms of Disease. By ROBERT CARSWELL, M.D. ib.
4. Teoria della Flogosi, di GIOVANNI RASORI . . . . ib.
Theory of Inflammation. By J. RASORI.

ART. VI.—Guy's Hospital Reports. Nos. VI. and VII. . . . 443

ART. VII.—Bemerkungen über den Brand der Kinder. V. Dr. A. LEOPOLD RICHTER 470

Observations on Infantile Gangrene. By Dr. A. L. RICHTER.

ART. VIII.—A Dialogue between a Bilious Patient and a Physician. By Dr. HENRY 476

ART. IX.—Essays on the most important Diseases of Women. By Dr. FERGUSON 482

ART. X.—Observationes Anatomicæ et Microscopicæ de Systematis Nervosi Structura. Auctore ROBERTO REMAK, Med. et Chir. Dr. . . . 500
Anatomical and Microscopical Observations on the Structure of the Nervous System. By ROBERT REMAK, M.D.

ART. XI.—Lectures on Lithotomy, delivered at the New York Hospital, December, 1837. By ALEXANDER H. STEVENS, M.D. . . . 505

ART. XII.—Ueber die Pulsation in der Oberbauchgegend als begleitendes Symptom der Indigestion. Von Dr. C. HOHNBAUM . . . . 511
On Pulsation in the Epigastrium as an accompanying Symptom of Indigestion. By Dr. CHARLES HOHNBAUM

ART. XIII.—The Village Pastor's Surgical and Medical Guide: in Letters from an old Physician to a young Clergyman, his Son. By F. SKRIMSHIRE, M.D. . 513

ART. XIV.—Recherches Anatomiques sur l'Emphysème Pulmonaire. Par le Dr. LOMBARD . . . . . . . . 516
Anatomical Researches on Pulmonary Emphysema. By Dr. LOMBARD.

ART. XV.—An Address delivered at the Birmingham Royal School of Medicine and Surgery. By VAUGHAN THOMAS, B.D. . . . 520

### PART SECOND.—Bibliographical Notices.

ART. I.—Observations on certain Parts of the Animal Economy, inclusive of several Papers from the Philosophical Transactions, &c. By JOHN HUNTER, F.R.S. With Notes by RICHARD OWEN, F.R.S., &c. . . . 523

ART. II.—On the Objects and Mutual Relations of the Medical Sciences: an Introductory Address delivered at the Middlesex Hospital School of Medicine, at the opening of the Winter Session. By FRANCIS LEIGHTON, M.D. . 525

PAGE
ART. III.—1. A Dictionary of Practical Surgery, &c. By SAMUEL COOPER . 526
2. Lexicon Medicum, or Medical Dictionary, &c. By the late ROBERT HOOPER, M.D. F.L.S. Seventh Edition, corrected and enlarged. By KLEIN GRANT, M.D. ib.

ART. IV.—A Letter to the Inhabitants of Ceylon, on Vaccination. By J. KINNIS, M.D. 527

ART. V.—A Treatise on Neuralgia. By RICHARD ROWLAND, M.D. . . 529

ART. VI.—Ausfürliche Encyklopädie der gesammten Staatsarzneikunde. Von GEORG FRIEDERICH MOST . . . . . . 530
A Complete Encyclopædia of Medical Jurisprudence. By Dr. G. F. MOST.

ART. VII.—Flora Medica: a Botanical Account of all the more important Plants used in Medicine, in different Parts of the World. By J. LINDLEY, PH.D. F.R.S. 531

ART. VIII.—Handbuch der Reagentien-und Zerlegungslehre, &c. Von Hofrath Dr. DU MENIL . . . . . . 533

Manual of Chemical Tests, and of Chemical Decomposition. By Dr. DU MENIL.

ART. IX.—A Letter to Dr. CHAMBERS, on the Nature and proper Treatment of Gout. By SIR CHARLES SCUDAMORE, M.D. F.R.S., &c. . . 534

ART. X.—De Cartilaginum Articularium ex Morbis Mutationes. Auct. L. H. SCHUMER, Jr. . . . . . . 535
On the Pathology of the Articulating Cartilages. By L. H. SCHUMER, M.D.

ART. XI.—On the successful Treatment of Consumptive Disorders, and Female Complaints connected therewith; on Scrofulous Diseases; and on the Management of delicate Health by Diet and Regimen. By J. J. FURNIVALL, M.D. 536

ART. XII.—Illustrations of Osteology. By THEODORE S. G. BOISRAGON, M.D. . 537

ART. XIII.—An Essay on Food, &c. By W. GRISENTHWAITE . . 538

ART. XIV.—Prostitution in London, &c. By MICHAEL RYAN, M.D. . . 539

ART. XV.—Observations on Homœopathy and Animal Magnetism, &c. By JAMES LOMAX BARDSLEY, M.D. F.L.S. . . . . . 540

---

### PART THIRD.—Selections from the British and Foreign Journals.

### I. THE FOREIGN JOURNALS.

#### ANATOMY AND PHYSIOLOGY.

Dr. VOLKMANN on the Fibres of the Spinal Marrow, &c. in the Rana Esculenta . 541
Dr. ROMBERG's case of Anæsthesia in the course of distribution of the Fifth Nerve 544
Professor BISCHOFF on Transfusion of Blood . . . . 548
J. C. G. FRICKE on the Temperature of the Vagina and Uterus before and during Menstruation, and of the Vagina during Pregnancy . . . 549

#### MEDICINE.

RASPAIL on Camphor, as a Panacea . . . . . . ib.
Dr. SCHWABE on the Character and Origin of the Pustula Maligna . . 550
M. GERARDIN on the Treatment of Quinsy by Scarification . . . ib.
Dr. BIESKE's case of Fatal Inflammation of the Vermiform Process . . 551
M. BRIQUET on the Local Employment of Mercury in Variolous Eruptions . 552
Prof. SEBASTIAN on the Origin, Growth, and Termination of Pulmonary Tubercle . 553

#### SURGERY.

M. MALGAIGNE on the Varieties and Treatment of Fractures of the Ribs. . 554

Dr. SPÖRER's case of Tetanus, with Trismus, successfully treated . . 559

Prof. DIEFFENBACH on the Division of the Sterno-cleido-mastoid Muscle . . 560

#### MIDWIFERY.

Dr. BAMBERGER's case of Pregnancy of the Fallopian Tube . . . 562

Dr. FRICKE's two cases of Episioraphy, to elucidate a Modification of the operation . ib.

TOXICOLOGY. PAGE

Dr. Deville's case of Poisoning by Arsenious Acid successfully treated by the Hydrated Tritoxide of Iron . . . . . 563
Dr. Berndt's Toxicological Experiments with Preparations of Chromium . 564

CHEMISTRY.

Prof. Sebastian on the Pathological Alterations in the Constitution of Bones . 566

MATERIA MEDICA.

Prof. Sementini on the Medicinal Properties of the Gray Oxide of Zinc . 567

MEDICAL STATISTICS.

Revaccination. 1. Results of the Revaccination of the Prussian Army in 1837.
2. Discussions in the French Academy. . . . . ib.
Influence of Artificial Feeding upon the Mortality of Infants . . 569

II. THE AMERICAN AND COLONIAL JOURNALS.

MEDICINE.

Dr. Bigelow's Remarks on Pneumothorax, &c. . . . . 569

PHARMACY.

Effervescent Magnesia . . . . . . 570

MIDWIFERY.

Obstetrical Enormity . . . . . . ib.

III. THE BRITISH JOURNALS.

ANATOMY AND PHYSIOLOGY.

Mr. Goodsir Junior on the Origin and Development of the Pulps and Sacs of the Human Teeth . . . . . 571
Dr. Lonsdale's Experimental Enquiry into the Physiological Action, the Poisonous Properties, and the Therapeutic Effects of the Hydrocyanic Acid . 572
Dr. Davy on a peculiarity of Structure in the Basilar Artery of Man . . 574
on the relative Prevalence of certain Organic Diseases . . 575

MEDICINE.

Dr. Greene on Tubercles in the Brain of Children . . . 576
Mr. Clarke on an Attempt to cure Elephantiasis by the poison of the Rattlesnake 578
Mr. Hutchinson on Revaccination at the Foundling Hospital . . 581
Dr. Law's Observations on the Exhibition of Mercury in minute Doses . . ib.
Dr. Greene on the Nervous Tremor of Children . . . 583

SURGERY.

Mr. Smee on the Formation of Moulding Tablets for Fractures, &c. . . ib.
Mr. Wilson on a Case of Hernia successfully treated on Dr. O'Beirne's plan . 584
Mr. Roberts on an astounding Injury of the Brain recovered from . . 585
Dr. Selwyn on Encysted Dropsy of the Thyroid Gland, with a method of Operation and Cure . . . . . . . 586

MIDWIFERY.

Mr. Owens on an enormous Weight of a Child at Birth . . . ib.

ANIMAL CHEMISTRY.

Dr. Rainy on Detection of Urea in the Blood of a [illegible]t who died of Cholera . ib.

NATURAL HIST[illegible]Y.

Dr. Bellingham on an Undescribed Species of Human Intestinal Worm . 587

Part Fourth.—Medical Intelligence.

The Office, Duties, and Qualifications of Coroners . . . 589
Officers of Health in the French Provinces . . . . 590
Medical Fees in America . . . . . . 591
Prof. Rau on the Mortality of Infants in the first year of Existence . . ib.
Books received for Review . . . . . 594

Index, Title, &c.

